# The Microwave Rotational Electric Resonance (RER) Spectrum of Benzothiazole

**DOI:** 10.3390/molecules28083419

**Published:** 2023-04-13

**Authors:** Hamza El Hadki, Kenneth J. Koziol, Oum Keltoum Kabbaj, Najia Komiha, Isabelle Kleiner, Ha Vinh Lam Nguyen

**Affiliations:** 1 Laboratory of Spectroscopy, Molecular Modeling, Materials, Nanomaterials, Water, and Environment, Faculty of Sciences, Mohammed V University, Av Ibn Battouta, Rabat B.P. 1014, Morocco; 2 Univ Paris Est Creteil and Université Paris Cité, CNRS, LISA, 94010 Créteil, France; kenneth.koziol@lisa.ipsl.fr; 3 Université Paris Cité and Univ Paris Est Creteil, CNRS, LISA, 75013 Paris, France; 4 Institut Universitaire de France (IUF), 75231 Paris, France

**Keywords:** microwave spectroscopy, quantum chemistry, nuclear quadrupole coupling, benzothiazole

## Abstract

The microwave spectra of benzothiazole were measured in the frequency range 2–26.5 GHz using a pulsed molecular jet Fourier transform microwave spectrometer. Hyperfine splittings arising from the quadrupole coupling of the ^14^N nucleus were fully resolved and analyzed simultaneously with the rotational frequencies. In total, 194 and 92 hyperfine components of the main species and the ^34^S isotopologue, respectively, were measured and fitted to measurement accuracy using a semi-rigid rotor model supplemented by a Hamiltonian accounting for the ^14^N nuclear quadrupole coupling effect. Highly accurate rotational constants, centrifugal distortion constants, and ^14^N nuclear quadrupole coupling constants were deduced. A large number of method and basis set combinations were used to optimize the molecular geometry of benzothiazole, and the calculated rotational constants were compared with the experimentally determined constants in the course of a benchmarking effort. The similar value of the *χ_cc_* quadrupole coupling constant when compared to other thiazole derivatives indicates only very small changes of the electronic environment at the nitrogen nucleus in these compounds. The small negative inertial defect of −0.056 uÅ^2^ hints that low-frequency out-of-plane vibrations are present in benzothiazole, similar to the observation for some other planar aromatic molecules.

## 1. Introduction

**Figure 1:**
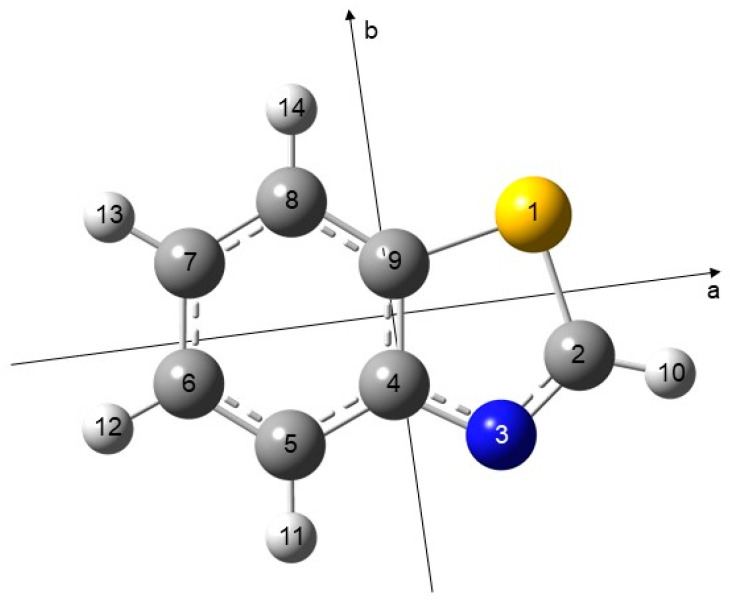
Molecular structure of benzothiazole optimized at the MP2/6-311++G(d,p) level of theory in the principal axes of inertia (*a*, *b*, and *c*), with a planar geometry. The *c*-axis (not shown) is perpendicular to the *ab*-plane. The nitrogen atom is blue and the sulfur atom is yellow.

Benzothiazole (BTA), see Figure 1, is a planar bicyclic compound in which a benzene ring is fused to a thiazole ring. It is a compound with wide industrial applications, such as in the synthetic rubber vulcanization, which exhibits an acute toxicity. BTA is also a respiratory irritant and dermal sensitizer [1]. Its derivative, 2-mercaptobenzothiazole, has been reported to be a rodent carcinogen [1]. BTA may volatilize from crumb rubber, resulting in inhalation exposure [2]. It is also present in clothing textiles, which are a potential source of environmental pollutants [3]. At the same time, its derivatives have attracted continuous interest due to their various biological activities, such as anticancer [4], antimicrobial [5], antimalarial [6], anti-inflammatory [7], and antidiabetic [8] activities. BTA derivatives were also evaluated as prospective diagnostic agents for amyloid binding in neurodegenerative diseases [9,10] and as histamine H3 antagonists [11]. The wide range of biological effects associated with this scaffold renders the structure of the core molecule BTA to be important and interesting. Among many molecular spectroscopic techniques, Fourier transform microwave spectroscopy is particularly useful for gas phase structural studies at the molecular level, allowing the determination of accurate geometry parameters without perturbation from matrix or solvent environments. The molecular structure, i.e., the mass distribution of atoms in the molecules, is directly linked to the microwave transition frequencies through the rotational constants.

Coupled with supersonic expansion through a pulsed nozzle with an inert carriergas such as helium, neon, or argon, a cooling of the vibrational and rotational states down to a rotational temperature of about 2 K is produced by the strong collisional regime during the adiabatic expansion, making only the rotational transitions in the vibrational ground state observable. The sensitivity of a Fourier transform microwave setup enables also the detection of minor isotopologues, such as ^34^S and ^13^C, in some cases, even ^15^N and ^18^O, in natural abundances of about 4.2%, 1.1%, 0.35%, and 0.21%, respectively. Since different isotopologues feature different total molecular masses, changes in isotopic substitution lead to different mass distributions and, as a consequence, distinct rotational constants. If the spectra of such minor isotopologues are available, the deduced rotational constants can be used to determine experimental atomic positions, bond lengths, and bond angles.

Nowadays, Fourier transform microwave spectroscopy is often integrated with quantum chemical calculations that serve as a powerful support for the spectral assignment. They deliver predicted values of the rotational constants to guide the assignment. For medium-sized or large molecules, or in cases where a sufficient number of minor isotopologue spectra cannot be observed, leading to the lack of an experimental microwave structure determination, indirect information on the molecular geometry through the rotational constants can be obtained by comparing them with the predicted ones. In the previous decades, not only more computational resources have become available during the information revolution, but also the accuracy of quantum chemical calculations has been significantly increased, and is still improving continuously with newer and more elaborate theoretical conceptualizations. Several benchmarking studies involving tight collaborations between microwave spectroscopic and theoretical groups have been successfully established, aiming to predict the experimental rotational constants with even higher accuracy [12,13,14,15,16,17]. On the one hand, this joint-venture allows experimentalists to efficiently assign the microwave spectra and to access detailed molecular geometries in the cases when minor isotopologue spectra are lacking. On the other hand, highly accurate constants obtained from the experiments can be utilized in return to benchmark results from quantum chemistry, helping theoreticians to identify methodological bottlenecks and limitations, and to push forward new methodology developments.

Using the combination of microwave spectroscopy and quantum chemical calculations, not only precise information about the molecular structures can be obtained. If an atom with a nuclear quadrupole moment is present in the molecule, information on the molecular electron density at this atom is also accessible. In a classical and idealized model, the nucleus of an atom is viewed as a monopole with a homogeneous charge distribution in a spherical symmetry. This is correct for nuclei with a spin of 0 and ½. For spins equal or higher than the absolute value of 1, the charge distribution is asymmetric in the shape of an ellipsoid, which creates an electric field gradient at the nucleus. BTA features a ^14^N nucleus with a nuclear spin *I* = 1. The nuclear quadrupole coupling with the molecular angular momentum *J* occurs for all nuclei with a nuclear spin *I* > ½. It is an interaction between the nuclear quadrupole moment arising from the non-spherical distribution of a nuclear charge and the electronic field gradient arising from the non-spherical distribution of the electronic charge around the nucleus. Although the effects of the molecular rotation towards the electric field gradients of the nuclei are minor, it is sufficient to result in hyperfine splittings of the rotational levels observable in the high-resolution microwave spectrum from which the nuclear quadrupole coupling constants can be determined. By comparing their values with those obtained for other thiazole derivatives, we can estimate the influence of substituents on the nuclear quadrupole coupling constants.

We present here the experimental results from a microwave spectroscopic investigation on BTA which aims at determining and understanding its molecular structure and the ^14^N nuclear quadrupole coupling effects. State-of-the-art quantum chemical calculations with an extensive benchmarking of several *ab initio* and density functional theory methods as well as Pople and Dunning basis sets were performed to support the experimental finding.

## 2. Quantum Chemical Calculations

### 2.1. Geometry Optimizations

Similar to thiazole and benzene, BTA possesses a planar, aromatic structure due to the delocalization of the π-electrons. Therefore, a single conformer is possible, as illustrated in Figure 1. To obtain the molecular geometry of BTA and to benchmark the rotational constants, full geometry optimizations were performed with the Gaussian 16 package [18] using various combinations of methods and basis sets in a systematic way. We employed two *ab initio* methods MP2 [19] and coupled cluster single and double excitation CCSD [20] as well as several exchange–correlation functionals from density functional theory method, such as B3LYP [21,22], Truhlar’s M06-2X [23], Head-Gordon’s ωB97X-D [24], Perdew–Burke–Ernzerhof PBE [25], and Yu’s Minnesota MN15 [26]. Furthermore, the B3LYP functional was combined with D3 [27], Becke–Johnson damping (D3BJ) [28], and with the Coulomb attenuation method (CAM-B3LYP) [29]. A total of 21 basis sets have been associated, such as the Pople’s split-valence double-zeta 6-31G and split-valence triple-zeta 6-311G basis sets [30] incorporating or not diffuse functions (+ or ++). Polarization functions of the types (d,p), (df,dp), (2d,2p), (2df,2pd), and (3df,3dp) are always included. In addition, Dunning’s double zeta polarization (cc-pVDZ) and triple zeta polarization (cc-pVTZ) basis sets are used [31] with or without diffuse functions (aug). Note that the basis set variation is not performed with the computationally expensive CCSD method, and we retain here with only the cc-pVDZ basis set. Vibrational harmonic frequencies were calculated on the optimized geometries to verify whether the stationary point is a saddle point or a true minimum geometry. The obtained rotational constants are presented in Table S1 of the Supplementary Materials. A selection of the results at three levels of theory, MP2/6-311++G(d,p), MP2/6-31G(d,p), and B3LYP-D3BJ/6-311++G(d,p) can be found in Table 1. The molecular structure optimized at the MP2/6-311++G(d,p) level is shown in Figure 1; the atomic Cartesian coordinates of this structure are available in Table S2.

### 2.2. ^14^N Nuclear Quadrupole Coupling Constants

The relation between the components of the electric field gradient tensor *q_ij_* (*i*, *j* = *a*, *b*, *c*) with those of the nuclear quadrupole coupling tensor *χ_ij_* is described by the following equation:

*χ_ij_* = (*eQ*/*h*) · *q_ij_* (1)

where *e* is the fundamental electric charge, *Q* is the electric quadrupole moment of the nucleus, and *h* is the Planck’s constant. Calculated nuclear quadrupole coupling constants can be obtained directly from geometry optimizations, as shown exemplarily for the diagonal elements *χ_aa_*, *χ_bb_*, and *χ_cc_* at three levels of theory in Table 1.

Several studies have indicated that the cost-efficient, semi-experimental method proposed by Bailey [32] for the calculations of ^14^N nuclear quadrupole coupling constants is reliable, especially for molecules containing conjugated double bonds [33,34,35,36,37,38]. Therefore, we applied this method to calculate the nuclear quadrupole coupling constants of BTA. With Bailey’s method, experimental data are calibrated at a chosen level of molecular orbital calculation. The experimental nuclear quadrupole coupling constants *χ_ij_* of 150 molecules are plotted against the electric field gradients *q_ij_*, showing a clear linear regression where the slope, *eQ/h* (see Equation (1), is 4.599(12) MHz/a.u. For BTA, after the molecular geometry had been optimized at a given level of theory (the MP2/6-311++G(d,p) level is often chosen, but other levels can be used also), the electric field gradient tensor at the ^14^N nucleus site was calculated at the B3PW91/6-311+G(d,p) level, which is recommended for modeling π-electron conjugate systems [38]. The quadrupole coupling tensor is directly proportional to the electric field gradient tensor by the calibration factor *eQ*/*h* = −4.599 MHz/a.u. determined from the linear regression. The nuclear quadrupole coupling constant values obtained with this method are *χ_aa_* = 1.727 MHz, *χ_bb_* = −4.036 MHz, *χ_cc_* = 2.309 MHz, *χ_ab_* = 0.078 MHz, *χ_ac_* = 0.000 MHz, and *χ_bc_* = 0.000 MHz. Note that for a planar molecule, the values of *χ_ac_* and *χ_bc_* should be zero.

## 3. Microwave Spectroscopy

The microwave spectra of BTA were measured using a molecular jet Fourier transform microwave spectrometer with a Fabry–Perot type resonator [39]. It operates between 2–26.5 GHz and is a modified version of that described in Ref. [40]. Therefore, details of the spectrometer will not be repeated here. BTA was purchased from Lancaster at a purity of 97% and was used as received. The sample was placed into a heated reservoir [41] at a temperature of 150 °C under helium gas stream. The total helium pressure was set to 2 bar. The helium-BTA mixture was expanded into the cavity at a rotational temperature of 2 K through a pulsed nozzle which reached a temperature of 140 °C. During the adiabatic expansion, the Doppler line and collisional broadening are simultaneously reduced by velocity equilibration, inducing strong rovibrational cooling. As a consequence, only the lowest rovibrational levels are populated which simplifies considerably the observed spectra. All spectra are measured with reference to a GPS disciplined rubidium frequency standard. The experimental uncertainty of the instrument is estimated to be 2 kHz for unblended lines [42].

**Figure 2:**
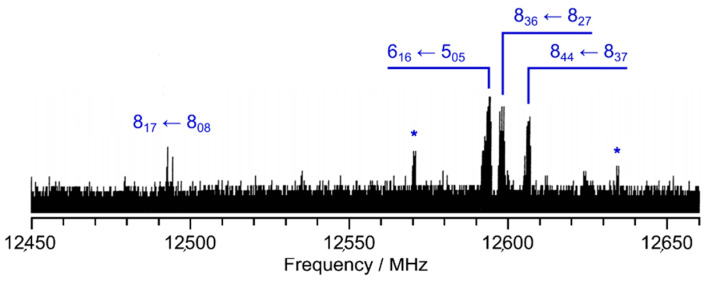
A portion of the survey spectrum of BTA in the frequency range from 12,450 to 12,660 MHz, showing hyperfine splittings arising from the ^14^N nuclear quadrupole coupling. Assigned lines are labelled by their rotational quantum numbers ${J^{\prime \prime }}_{K_{a}^{\prime \prime }K_{c}^{\prime \prime }}\leftarrow {J^{\prime }}_{K_{a}^{\prime }K_{c}^{\prime }}$ and all belong to the main isotopologue of BTA. Lines marked with an asterisk are from impurities, as they disappeared when being remeasured later, probably due to a higher vapor pressure than that of BTA.

**Figure 3:**
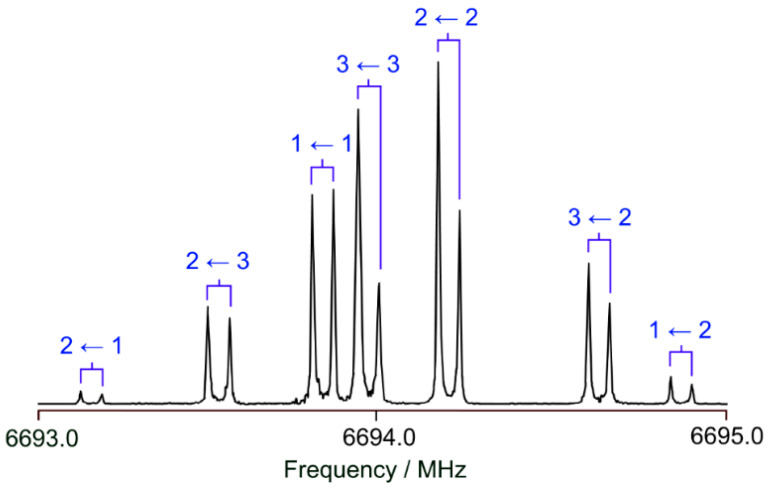
A typical spectrum of the *b*-type 2_21_ ← 2_12_ transition of BTA, featuring the ^14^N quadrupole hyperfine structure with the hyperfine components indicated as *F″* ← *F′*. The Doppler pairs arising from the coaxial arrangement between the resonators and the molecular beam are connected by brackets. The intensities are not reliable since hyperfine components close to the polarization frequency (at 6694.0 MHz) appear to be more intense.

At the beginning, a survey spectrum was recorded. A portion of the spectrum in the frequency range from 12,450 to 12,660 MHz is illustrated Figure 2. High-resolution measurements were recorded for each signal found in the survey spectrum to determine the exact frequencies. Due to the coaxial arrangement of the resonators and the molecular beam, all rotational transitions appear as doublets arising from the Doppler effect. The expected hyperfine splittings due to the ^14^N nuclear quadrupole coupling could be observed. Due to this hyperfine structure, most rotational transitions appear as a triplet (the three strongest *F*-components). For some transitions, i.e., the transition shown in Figure 3, it was possible to measure all theoretically possible quadrupole *F*-components. A total of 194 lines with *J* ≤ 12 and *K_a_* ≤ 4 were measured and fitted using the program *XIAM* [43] to precisely determine the rotational constants *A*, *B*, and *C*, the quartic centrifugal distortion constants in the A-reduced form Δ*_J_*, Δ*_K_*, Δ*_JK_*, *δ_J_*, and *δ_K_* of Watson’s asymmetric rotor Hamiltonian, as well as the diagonal elements *χ_aa_*, *χ_bb_*, and *χ_cc_* of the nuclear quadrupole coupling tensor of the ^14^N nucleus. The off-diagonal quadrupole coupling constants could not be determined, which is often the case for ^14^N nuclear quadrupole coupling due to the relatively small quadrupole moment of the ^14^N nucleus.

To obtain information on the structure of BTA, we first searched for the spectra of the ^34^S isotopologue in natural abundance of about 4%. The search was straightforward and we could measure and fit the frequencies of 92 transitions also using the rotational constants *A*, *B*, and *C*, the quartic centrifugal distortion constants Δ*_J_*, Δ*_K_*, Δ*_JK_*, *δ_J_*, and *δ_K_*, and the nuclear quadrupole coupling constants *χ_aa_*, *χ_bb_*, and *χ_cc_* of the ^14^N nucleus. The obtained parameters are summarized in Table 2 along with those of the parent species fit. The assigned frequencies along with their residues are listed in Table S3 in the Supplementary Materials for both the parent and the ^34^S species. We then searched for the spectra of the ^13^C isotopologues but their transitions were too weak to be observed. Because the natural abundance of ^15^N is even lower than that of ^13^C, the spectrum of the ^15^N isotopologue should be even weaker and, therefore, was not searched.

## 4. Results and Discussion

The *XIAM* fit containing 194 hyperfine components of 52 rotational transitions reached a standard deviation of 1.6 kHz, which is within the experimental accuracy of 2 kHz of the spectrometer in use. All fitted molecular parameters such as the rotational constants *A*, *B*, and *C*, the quartic centrifugal distortion constants Δ*_J_*, Δ*_K_*, Δ*_JK_*, *δ_J_*, and *δ_K_*, and the ^14^N nuclear quadrupole coupling constants *χ_aa_*, *χ_bb_*, and *χ_cc_* are determined with high accuracy. The experimentally deduced values are close to the calculated ones. The quartic centrifugal distortion constants are extremely well predicted, as can be seen in Table 2. This is probably due to the rigidity of the aromatic ring. Bailey’s method using the electric field gradient calibration at the B3PW91/6-311+G(d,p) level maintains its reliability in calculating nuclear quadrupole coupling constant values for aromatic ring containing molecules. The *χ_aa_*, *χ_bb_*, and *χ_cc_* values obtained with this method are significantly closer to the experimental values than those calculated directly from geometry optimizations shown in Table 1. For the rotational constants, the values obtained from the equilibrium structure *A*_e_, *B*_e_, and *C*_e_ are closer to the experimental values *A*_0_, *B*_0_, and *C*_0_ obtained at the vibrational ground state than the calculated *A*_0_, *B*_0_, and *C*_0_ values from anharmonic frequency calculations (see Table 1). This is most probably due to error compensation. From theoretical perspective, calculated ground state rotational constants should be more accurate, and it is not physically meaningful to compare experimental *A*_0_, *B*_0_, and *C*_0_ constants with calculated *A*_e_, *B*_e_, and *C*_e_ constants. Practically, cost-efficient geometry optimizations compared to resource-intense anharmonic frequency calculations, in combination with accidental accuracy from error compensation, renders the use of *A*_e_, *B*_e_, and *C*_e_ constants to be helpful in assigning microwave spectra. With the continuous increase in computational capacity, benchmarking has become a core activity in the development of quantum chemical methods. A number of structure optimizations with different methods and basis sets described in Section 2.1. were performed for benchmarking purposes by comparing the calculated rotational constants *A*_e_, *B*_e_, and *C*_e_ of BTA to the accurately determined experimental constants *A*_0_ = 3174.630260(98) MHz, *B*_0_ = 1341.764826(67) MHz, and *C*_0_ = 943.232720(28) MHz. We found that for all levels of theory, the calculated equilibrium rotational constants are quite close to those obtained experimentally with a deviation of about 1% or less. Although some levels performed better than some others, there is no clue to conclude a “golden level” for BTA. We note that the three levels mentioned in Table 1 all yielded good results, and among them the MP2/6-31G(d,p) level performed best, as it did in the cases of many other molecules containing aromatic rings such as coumarin [44], quinoline and isoquinoline [45], 2-ethylfuran [46], *o*-methylanisole [47], and a series of acetylmethylthiophene [13,48,49].

Though the number of lines assigned for the ^34^S isotopologue is only half as many as for the parent species, the ^34^S fit is also very satisfactory with standard deviation close to the measurement accuracy of 2 kHz [42] and the fitted parameters are well determined. Since only rotational constants of the ^34^S isotopologue are available, a complete structure determination cannot be performed but the position of the sulfur atom can be calculated utilizing Kraitchman’s equations [50] with errors estimated with Costain’s rule [51]. The sulfur coordinates with respect to the center of mass are |*a*_0_| = 1.74823(86) Å, |*b*_0_| = 0.9055(17) Å, and *c* = 0 Å (fixed value due to molecular symmetry), which are in very good agreement with the values obtained from the geometry calculated at the MP2/6-31G(d,p) level (*a*_e_ = 1.757 Å, *b*_e_ = 0.903 Å, and *c*_e_ = 0.000 Å, see Table S2 of the Supplementary Materials). The signs of the coordinates cannot be obtained from Kraitchman’s equations and should be taken from *ab initio* according to the axis orientations shown in Figure 1. The very small differences between the experimental and the theoretical coordinates can be, again, explained by the fact that the experimental values *a*_0_ and *b*_0_ refer to the vibrational ground state and the theoretical values *a*_e_ and *b*_e_ to the equilibrium structure. We then corrected the experimental *A*_0_, *B*_0_, and *C*_0_ rotational constants of the parent species and the ^34^S isotopologue with the rovibrational corrections obtained from the cubic force field calculated at the MP2/6-31G(d,p) level. The resulted “semi-experimental equilibrium substitution” sulfur coordinates are $\left|a_{e}^{\mathrm{SE}}\right|$ = 1.74656(86) Å and $\left|b_{e}^{\mathrm{SE}}\right|$ = 0.9024(17) Å, in excellent agreement to the equilibrium values.

**Figure 4:**
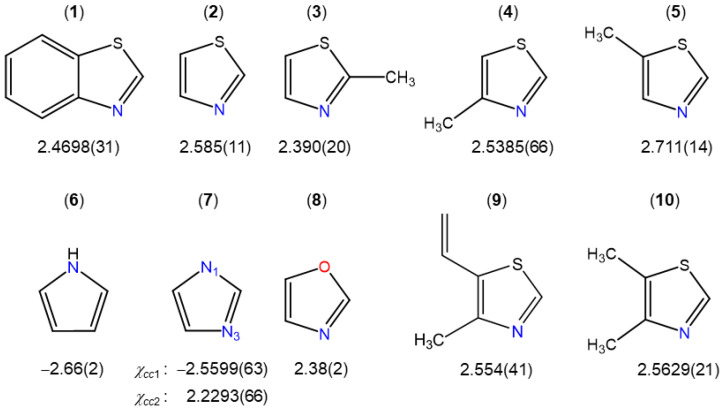
*χ_cc_* values in some thiazole derivatives and aromatic five-membered rings (in MHz). (**1**) Benzothiazole (this work), (**2**) thiazole [52], (**3**) 2-methylthiazole [53], (**4**) 4-methylthiazole [54], (**5**) 5-methylthiazole [55], (**6**) pyrrole [56], (**7**) imidazole [57], (**8**) oxazole [58], (**9**) 4-methyl-5-vinylthiazole [37], and (**10**) 4,5-dimethylthiazole [59].

To compare the experimental ^14^N nuclear quadrupole coupling constant values of BTA and, consequently, the field gradient tensor at the site of the nitrogen nucleus with other thiazole derivatives, we selected some planar molecules, as illustrated in Figure 4. Due to planarity, the *c*-principal axis and one principal axis of the nitrogen coupling tensor are collinear, since they are both perpendicular to the plane of symmetry (the ring plane). Therefore, we can directly compare the *χ_cc_* values. 

Among all thiazole derivatives, i.e., BTA (**1**) (this work), thiazole (**2**) [52], 2-methylthiazole (**3**) [53], 4-methylthiazole (**4**) [54], 5-methylthiazole (**5**) [55], 4-methyl-5-vinylthiazole (**9**) [37], and 4,5-dimethylthiazole (**10**) [59], though all *χ_cc_* values are similar, a small variation from 2.390 MHz in 2-methylthiazole (**3**) to 2.711 MHz in 5-methylthiazole (**5**) can be observed, showing that the electronic environment at the nitrogen nucleus changes slightly despite the fact that the molecules are very similar. It is also interesting that (i) the *χ_cc_* value found for oxazole (**8**) [58] is almost the same as that of 2-methylthiazole (**3**) [53], and (ii) the value of the nitrogen atom at the 1-position of pyrrole (**6**) [56] and imidazole (**7**) [57] is negative due to a different bond situation at the ^14^N atom. To form one N–H bond and two N–C bonds, three of five ^14^N electrons are involved in three sp^2^ orbitals, and the other two electrons occupy the p orbital and contribute to the π electron system. Altogether, the nitrogen atoms donate two electrons to the aromatic system, while the others featuring positive *χ_cc_* values provide only one electron. There, two ^14^N electrons are used in two sp^2^ orbitals to form two bonds, while the electron lone pair occupies the third sp^2^ orbital, leaving only one electron in the p orbital. If we consider that *χ_cc_* is related to the unbalanced electrons in a p_z_ orbital, the positive values found for BTA as well as other thiazole derivatives illustrated in Figure 4 also show that for these molecules there is an excess of electron density along the *z* axis of the ^14^N nucleus [60].

As an aromatic molecule with a π-conjugated system fused by thiazole and benzene, there is no doubt on the planarity of BTA, even though a complete experimental structure determination of the heavy atom backbone is not possible due to the lack of all ^13^C isotopologue spectra and the ^15^N isotopologue spectrum. A well-known judgement for the planarity of a molecule is its small positive inertial defect value, as observed for, e.g., thiazole (0.074 uÅ^2^) [52], oxazole (0.056 uÅ^2^) [58], pyrrole (0.017 uÅ^2^) [56], and 2-thiophenecarboxaldehyde (0.008 uÅ^2^) [61]. The inertial defect *Δ_c_* = *I_c_* – *I_a_* – *I_b_* of –0.056 uÅ^2^ found for BTA is very close to zero, but negative. In many molecules whose molecular structures were experimentally determined to be planar such as coumarin [44] and 1,4-naphthoquinone [62], a negative value of the inertial defect was found due to low-frequency out-of-plane vibrations. This was discussed in detail by Oka [63]. The negative value varies, and the largest ones ranging from –0.6 uÅ^2^ to –0.8 uÅ^2^ were found for styrene and its halogenated derivatives. The very small, but negative inertial defect of BTA might hint that such vibrations are also present in the molecule. The value of –0.082 uÅ^2^ obtained from rotational constants calculated with the anharmonic force field is also negative and very close to the experimental value.

## 5. Conclusions

The microwave spectrum of BTA was measured in the frequency range between 2 and 26.5 GHz, aiming at determining precisely its molecular geometry parameters. All rotational transitions of the rigid rotor feature hyperfine splittings arising from the quadrupole coupling of the ^14^N nucleus. A large data set with 194 hyperfine components was included in a fit where geometry parameters (the *A*, *B*, and *C* rotational constants and the Δ*_J_*, Δ*_K_*, Δ*_JK_*, *δ_J_*, and *δ_K_* quartic centrifugal distortion constants) are obtained together with the ^14^N diagonal nuclear quadrupole coupling constants *χ_aa_*, *χ_bb_*, and *χ_cc_*. The spectrum of the ^34^S minor isotopologue was also observed, including 92 transitions. By comparing the *χ_cc_* value of 2.4698(31) MHz of BTA to those of other thiazole derivatives, we found that though all *χ_cc_* values are similar, there is a small variation from 2.390 MHz to 2.711 MHz, showing that the electronic environment at the nitrogen nucleus is slightly different in those molecules. The very small, but negative inertial defect of −0.056 uÅ^2^ proposes that low-frequency out-of-plane vibrations are present in BTA.

## Figures and Tables

**Table 1:** The equilibrium *A*_e_, *B*_e_, and *C*_e_ and ground vibrational state *A*_0_, *B*_0_, and *C*_0_ rotational constants, ^14^N diagonal nuclear quadrupole coupling constants *χ_aa_*, *χ_bb_*, and *χ_cc_*, and dipole moment components *μ_a_*, *μ_b_*, and *μ_c_* of BTA obtained at the MP2/6-311++G(d,p), MP2/6-31G(d,p), and B3LYP-D3BJ/6-311++G(d,p) levels of theory.

		MP2/6-31G(d,p)	MP2/6-311++G(d,p)	B3LYP-D3BJ
*A*_e_	MHz	3166.6	3160.1	3168.6
*B*_e_	MHz	1338.7	1335.9	1334.2
*C*_e_	MHz	940.9	939.0	938.8
*A*_0_	MHz	3145.7	3136.4	3146.7
*B*_0_	MHz	1332.1	1328.2	1327.0
*C*_0_	MHz	936.0	933.1	933.5
*χ_aa_*	MHz	1.640	1.717	1.785
*χ_bb_*	MHz	−3.598	−3.922	−4.395
*χ_cc_*	MHz	1.967	2.205	2.611
|*μ_a_*|	D	0.37	0.24	0.33
|*μ_b_*|	D	1.33	1.56	1.36
|*μ_c_*|	D	0.00	0.00	0.00

**Table 2:** Molecular parameters of the main species and the ^34^S isotopologue of BTA obtained with the *XIAM* program by fitting the rotational constants *A*, *B*, and *C*, the quartic centrifugal distortion constants Δ*_J_*, Δ*_K_*, Δ*_JK_*, *δ_J_*, and *δ_K_*, as well as the diagonal nuclear quadrupole coupling constants *χ_aa_*, *χ_bb_*, *χ_cc_*.

Par. ^a^	Unit	^32^S *XIAM*	^32^S Calc ^b^	^34^S *XIAM*
*A*	MHz	3174.630260(98)	3160.13239	3143.66433(21)
*B*	MHz	1341.764826(67)	1335.93670	1320.53713(11)
*C*	MHz	943.232720(28)	938.98407	930.00014(6)
Δ*_J_*	kHz	0.02681(42)	0.025479	0.02678(49)
Δ*_JK_*	kHz	0.0472(22)	0.045033	0.0432(38)
Δ*_K_*	kHz	0.175(10)	0.17589	0.185(14)
*δ_J_*	kHz	0.00799(25)	0.007678	0.00754(30)
*δ_K_*	kHz	0.0696(39)	0.06822	0.0643(62)
*χ_aa_*	MHz	1.60208(92)	1.727 ^c^	1.5975(31)
*χ_bb_* ^d^	MHz	−4.0719(13)	−4.036 ^c^	−4.0686(34)
*χ_cc_* ^d^	MHz	2.4698(31)	2.309 ^c^	2.4710(71)
N ^e^		194	–	92
σ ^f^	kHz	1.6	–	2.4

^a^ All parameters refer to the principal axis system. Watson’s A reduction and I^r^ representation were used. Standard errors in parentheses are in the units of the last significant digits. ^b^ Calculated at the MP2/6-311++G(d,p) level of theory. The rotational constants *A*, *B*, and *C* are the equilibrium ones from geometry optimizations (see Table 1); the centrifugal distortion constants Δ*_J_*, Δ*_K_*, Δ*_JK_*, *δ_J_*, and *δ_K_* are from harmonic force field calculations. ^c^ Obtained with Bailey’s calibration [32] from electric field gradient calculations at the B3PW91/6-311+G(d,p) level of theory on the MP2/6-311++G(d,p) optimized structure with a correction factor *eQ*/*h* = −4.599 MHz/a.u. recommended for a molecule containing a conjugated double bond system [38]. ^d^ Derived from the fitted parameters *χ_aa_* and *χ_bb_*–*χ_cc_* = −6.5417(17) MHz. ^e^ Number of lines. ^f^ Standard deviation of the fit.

## Data Availability

Data are contained within the article and Supplementary Materials.

## Supplementary Materials

The following supporting information can be downloaded at: https://www.mdpi.com/article/10.3390/molecules28083419/s1, Table S1: The rotational constants *A*, *B*, *C* of BTA calculated at different levels of theory and their deviations to the experimental values; Table S2: Geometry parameters in the principal axes of inertia of BTA calculated at the MP2/6-31G(d,p), MP2/6-311++G(d,p), and B3LYP-D3BJ/6-311++G(d,p) levels of theory; Table S3: Calculated and observed frequencies of the main species and the ^34^S isotopologue of BTA.
