# Supplementary material for: The Microwave Rotational Electric Resonance (RER) Spectrum of Benzothiazole
Source: Molecules. 2023 Apr 13;28(8):3419. doi: 10.3390/molecules28083419 (PMC10146593; doi:10.3390/molecules28083419)
Supplement: Supplementary file 1 [file molecules-28-03419-s001.zip › molecules-2312669-supplementary.pdf]

Table S1. The rotational constants *A*, *B*, *C* (in MHz) of benzothiazole calculated at different levels of theory and their deviations to the experimental values (exp. − calc.) Δ*A*, Δ*B*, and Δ*C*, respectively (in MHz).

**Level of theory**	***A***	**Δ*A***	***B***	**Δ*B***	***C***	**Δ*C***
MP2/6-31G(d,p)	3166.6	8.0	1338.7	3.1	940.9	2.3
MP2/6-31+G(d,p)	3163.7	11.0	1335.9	5.9	939.3	4.0
MP2/6-31++G(d,p)	3163.6	11.0	1335.9	5.9	939.3	4.0
MP2/6-311G(d,p)	3161.7	12.9	1338.3	3.4	940.3	2.9
MP2/6-311+G(d,p)	3160.1	14.5	1335.9	5.8	939.0	4.3
MP2/6-311++G(d,p)	3160.1	14.5	1335.9	5.8	939.0	4.3
MP2/6-311G(2d,2p)	3168.5	6.2	1340.6	1.1	942.0	1.2
MP2/6-311+G(2d,2p)	3169.4	5.2	1339.5	2.3	941.6	1.7
MP2/6-311++G(2d,2p)	3169.4	5.2	1339.5	2.3	941.6	1.7
MP2/6-311G(df,pd)	3183.8	–9.2	1347.6	–5.8	946.8	–3.6
MP2/6-311+G(df,pd)	3181.1	–6.5	1345.3	–3.5	945.5	–2.2
MP2/6-311++G(df,pd)	3180.9	–6.3	1345.3	–3.5	945.4	–2.2
MP2/6-311G(2df,2pd)	3186.8	–12.2	1349.5	–7.7	948.0	–4.8
MP2/6-311+G(2df,2pd)	3186.6	–12.0	1347.9	–6.1	947.2	–4.0
MP2/6-311++G(2df,2pd)	3186.6	–12.0	1347.9	–6.1	947.2	–4.0
MP2/6-311G(3df,3pd)	3189.9	–15.3	1350.4	–8.7	948.8	–5.5
MP2/6-311+G(3df,3pd)	3188.8	–14.2	1349.5	–7.7	948.2	–5.0
MP2/6-311++G(3df,3pd)	3188.7	–14.1	1349.5	–7.7	948.2	–5.0
MP2/cc-pVDZ	3123.4	51.2	1324.9	16.9	930.3	13.0
MP2/aug-cc-pVDZ	3115.6	59.0	1321.5	20.3	927.9	15.3
MP2/cc-pVTZ	3179.9	–5.3	1346.3	–4.5	945.8	–2.6
MP2/aug-cc-pVTZ	3179.1	–4.4	1345.6	–3.8	945.4	–2.2
B3LYP-D3/6-31G(d,p)	3154.0	20.7	1330.6	11.2	935.8	7.5
B3LYP-D3/6-31+G(d,p)	3153.3	21.3	1328.5	13.3	934.7	8.5
B3LYP-D3/6-31++G(d,p)	3153.3	21.4	1328.5	13.2	934.7	8.5
B3LYP-D3/6-311G(d,p)	3165.9	8.8	1334.0	7.7	938.6	4.7
B3LYP-D3/6-311+G(d,p)	3166.3	8.3	1333.1	8.6	938.1	5.1
B3LYP-D3/6-311++G(d,p)	3166.2	8.4	1333.1	8.6	938.1	5.1
B3LYP-D3/6-311G(2d,2p)	3179.5	–4.9	1338.7	3.1	942.0	1.2
B3LYP-D3/6-311+G(2d,2p)	3180.5	–5.9	1337.9	3.8	941.8	1.5
B3LYP-D3/6-311++G(2d,2p)	3180.5	–5.9	1337.9	3.8	941.8	1.5
B3LYP-D3/6-311G(df,pd)	3177.3	–2.7	1338.1	3.7	941.6	1.7
B3LYP-D3/6-311+G(df,pd)	3178.0	–3.4	1337.2	4.6	941.2	2.1
B3LYP-D3/6-311++G(df,pd)	3178.0	–3.3	1337.2	4.6	941.2	2.1
B3LYP-D3/6-311G(2df,2pd)	3187.6	–12.9	1341.7	0.1	944.3	–1.0

B3LYP-D3/6-311+G(2df,2pd)	3188.4	–13.8	1340.9	0.9	943.9	–0.7
B3LYP-D3/6-311++G(2df,2pd)	3188.4	–13.8	1340.9	0.9	943.9	–0.7
B3LYP-D3/6-311G(3df,3pd)	3192.1	–17.5	1343.0	–1.2	945.3	–2.0
B3LYP-D3/6-311+G(3df,3pd)	3192.5	–17.9	1342.5	–0.8	945.1	–1.9
B3LYP-D3/6-311++G(3df,3pd)	3192.4	–17.8	1342.5	–0.8	945.1	–1.9
B3LYP-D3/cc-pVDZ	3142.5	32.1	1326.5	15.3	932.7	10.5
B3LYP-D3/aug-cc-pVDZ	3145.2	29.5	1326.0	15.8	932.7	10.5
B3LYP-D3/cc-pVTZ	3185.2	–10.6	1340.3	1.5	943.3	–0.1
B3LYP-D3/aug-cc-pVTZ	3186.0	–11.3	1340.2	1.6	943.4	–0.1
B3LYP-D3BJ/6-31G(d,p)	3156.9	17.8	1331.4	10.4	936.5	6.8
B3LYP-D3BJ/6-31+G(d,p)	3156.2	18.4	1329.4	12.4	935.4	7.8
B3LYP-D3BJ/6-31++G(d,p)	3156.2	18.5	1329.4	12.4	935.4	7.8
B3LYP-D3BJ/6-311G(d,p)	3168.2	6.4	1335.1	6.7	939.3	4.0
B3LYP-D3BJ/6-311+G(d,p)	3168.7	5.9	1334.2	7.6	938.9	4.4
B3LYP-D3BJ/6-311++G(d,p)	3168.6	6.0	1334.2	7.6	938.9	4.4
B3LYP-D3BJ/6-311G(2d,2p)	3182.2	–7.5	1339.4	2.4	942.6	0.6
B3LYP-D3BJ/6-311+G(2d,2p)	3182.6	–8.0	1338.9	2.8	942.4	0.8
B3LYP-D3BJ/6-311++G(2d,2p)	3182.6	–7.9	1338.9	2.8	942.4	0.8
B3LYP-D3BJ/6-311G(df,pd)	3180.0	–5.4	1338.8	3.0	942.2	1.1
B3LYP-D3BJ/6-311+G(df,pd)	3180.7	–6.0	1338.0	3.8	941.8	1.5
B3LYP-D3BJ/6-311++G(df,pd)	3180.6	–6.0	1338.0	3.8	941.8	1.5
B3LYP-D3BJ/6-311G(2df,2pd)	3190.1	–15.5	1342.4	–0.6	944.8	–1.6
B3LYP-D3BJ/6-311+G(2df,2pd)	3190.9	–16.3	1341.6	0.2	944.5	–1.3
B3LYP-D3BJ/6-311++G(2df,2pd)	3190.9	–16.3	1341.6	0.2	944.5	–1.3
B3LYP-D3BJ/6-311G(3df,3pd)	3194.6	–20.0	1343.6	–1.9	945.8	–2.6
B3LYP-D3BJ/6-311+G(3df,3pd)	3195.0	–20.4	1343.2	–1.5	945.7	–2.4
B3LYP-D3BJ/6-311++G(3df,3pd)	3194.9	–20.3	1343.2	–1.5	945.7	–2.4
B3LYP-D3BJ/cc-pVDZ	3145.6	29.1	1327.4	14.4	933.5	9.8
B3LYP-D3BJ/aug-cc-pVDZ	3148.2	26.4	1326.9	14.9	933.5	9.8
B3LYP-D3BJ/cc-pVTZ	3187.8	–13.2	1341.0	0.8	943.9	–0.7
B3LYP-D3BJ/aug-cc-pVTZ	3188.5	–13.9	1340.9	0.9	943.9	–0.7
M06-2X/6-31G(d,p)	3181.4	–6.7	1341.0	0.8	943.3	–0.1
M06-2X/6-31+G(d,p)	3180.0	–5.4	1339.4	2.3	942.5	0.8
M06-2X/6-31++G(d,p)	3180.0	–5.4	1339.4	2.3	942.5	0.8
M06-2X/6-311G(d,p)	3190.3	–15.7	1344.0	–2.2	945.6	–2.4
M06-2X/6-311+G(d,p)	3190.4	–15.8	1343.1	–1.4	945.2	–2.0
M06-2X/6-311++G(d,p)	3190.3	–15.7	1343.1	–1.4	945.2	–2.0
M06-2X/6-311G(df,pd)	3199.7	–25.1	1347.2	–5.5	948.1	–4.8
M06-2X/6-311+G(df,pd)	3200.1	–25.5	1346.4	–4.7	947.7	–4.5
M06-2X/6-311++G(df,pd)	3200.1	–25.4	1346.4	–4.7	947.7	–4.5
M06-2X/6-311G(3df,3pd)	3212.0	–37.3	1351.4	–9.7	951.2	–8.0

M06-2X/6-311+G(3df,3pd)	3212.0	–37.4	1351.1	–9.3	951.0	–7.8
M06-2X/6-311++G(3df,3pd)	3212.0	–37.4	1351.0	–9.3	951.0	–7.8
M06-2X/cc-pVDZ	3171.7	2.9	1337.8	4.0	940.9	2.3
M06-2X/aug-cc-pVDZ	3173.1	1.5	1337.4	4.4	940.8	2.4
M06-2X/cc-pVTZ	3207.1	–32.5	1349.2	–7.5	949.7	–6.5
M06-2X/aug-cc-pVTZ	3207.7	–33.1	1349.3	–7.6	949.8	–6.6
M06-2X-D3/6-31G(d,p)	3181.3	–6.7	1340.9	0.9	943.3	–0.1
M06-2X-D3/6-31+G(d,p)	3180.0	–5.3	1339.4	2.4	942.4	0.8
M06-2X-D3/6-31++G(d,p)	3179.9	–5.3	1339.4	2.4	942.4	0.8
M06-2X-D3/6-311G(d,p)	3190.2	–15.6	1344.0	–2.2	945.6	–2.4
M06-2X-D3/6-311+G(d,p)	3190.3	–15.7	1343.1	–1.3	945.2	–2.0
M06-2X-D3/6-311++G(d,p)	3190.3	–15.6	1343.1	–1.3	945.2	–1.9
M06-2X-D3/6-311G(2d,2p)	3201.8	–27.1	1348.0	–6.3	948.6	–5.4
M06-2X-D3/6-311+G(2d,2p)	3202.4	–27.8	1347.3	–5.6	948.3	–5.1
M06-2X-D3/6-311++G(2d,2p)	3202.4	–27.8	1347.3	–5.6	948.3	–5.1
M06-2X-D3/6-311G(df,pd)	3199.7	–25.0	1347.2	–5.4	948.0	–4.8
M06-2X-D3/6-311+G(df,pd)	3200.0	–25.4	1346.4	–4.6	947.7	–4.4
M06-2X-D3/6-311G(2df,2pd)	3209.1	–34.5	1350.5	–8.8	950.5	–7.3
M06-2X-D3/6-311+G(2df,2pd)	3209.5	–34.9	1349.8	–8.0	950.2	–6.9
M06-2X-D3/6-311++G(2df,2pd)	3209.5	–34.9	1349.8	–8.0	950.2	–6.9
M06-2X-D3/6-311G(3df,3pd)	3211.9	–37.3	1351.4	–9.6	951.2	–7.9
M06-2X-D3/6-311+G(3df,3pd)	3211.9	–37.3	1351.0	–9.3	951.0	–7.8
M06-2X-D3/6-311++G(3df,3pd)	3211.9	–37.3	1351.0	–9.2	951.0	–7.8
M06-2X-D3/cc-pVDZ	3171.6	3.0	1337.8	4.0	940.9	2.3
M06-2X-D3/aug-cc-pVDZ	3173.1	1.6	1337.3	4.4	940.8	2.4
M06-2X-D3/cc-pVTZ	3207.0	–32.4	1349.2	–7.4	949.7	–6.4
M06-2X-D3/aug-cc-pVTZ	3207.7	–33.0	1349.3	–7.5	949.8	–6.5
ωB97X-D /6-31G(d,p)	3194.5	–19.9	1344.1	–2.4	946.1	–2.8
ωB97X-D/6-31+G(d,p)	3194.9	–20.3	1343.2	–1.4	945.6	–2.4
ωB97X-D/6-31++G(d,p)	3194.8	–20.2	1343.2	–1.4	945.6	–2.4
ωB97X-D/6-311G(d,p)	3194.9	–20.3	1343.2	–1.4	945.6	–2.4
ωB97X-D/6-311+G(d,p)	3194.8	–20.2	1343.2	–1.4	945.6	–2.4
ωB97X-D/6-311++G(d,p)	3194.5	–19.9	1344.1	–2.4	946.1	–2.8
ωB97X-D/6-311G(2d,2p)	3207.1	–32.5	1348.6	–6.9	949.4	–6.2
ωB97X-D/6-311+G(2d,2p)	3208.0	–33.3	1347.9	–6.1	949.1	–5.9
ωB97X-D/6-311++G(2d,2p)	3207.9	–33.3	1347.9	–6.1	949.1	–5.9
ωB97X-D/6-311G(df,pd)	3204.6	–30.0	1347.8	–6.1	948.8	–5.6
ωB97X-D/6-311+G(df,pd)	3205.3	–30.7	1346.9	–5.2	948.4	–5.2
ωB97X-D/6-311++G(df,pd)	3205.2	–30.6	1346.9	–5.2	948.4	–5.2
ωB97X-D/6-311G(2df,2pd)	3214.6	–39.9	1351.4	–9.6	951.4	–8.2
ωB97X-D/6-311+G(2df,2pd)	3215.3	–40.6	1350.6	–8.9	951.1	–7.9

ωB97X-D/6-311++G(2df,2pd)	3215.3	–40.6	1350.6	–8.9	951.1	–7.9
ωB97X-D/6-311G(3df,3pd)	3218.5	–43.9	1352.6	–10.9	952.4	–9.1
ωB97X-D/6-311+G(3df,3pd)	3218.9	–44.2	1352.2	–10.5	952.2	–9.0
ωB97X-D/6-311++G(3df,3pd)	3218.8	–44.2	1352.2	–10.5	952.2	–9.0
ωB97X-D/cc-pVDZ	3171.5	3.1	1336.4	5.4	940.2	3.0
ωB97X-D/aug-cc-pVDZ	3173.8	0.9	1336.1	5.7	940.3	3.0
ωB97X-D/cc-pVTZ	3212.5	–37.8	1350.2	–8.5	950.7	–7.4
ωB97X-D/aug-cc-pVTZ	3213.3	–38.7	1350.2	–8.5	950.7	–7.5
PBE0/6-31G(d,p)	3124.3	50.3	1322.9	18.9	929.4	13.9
PBE0/6-31+G(d,p)	3123.9	50.7	1321.1	20.6	928.5	14.8
PBE0/6-31++G(d,p)	3123.9	50.7	1321.2	20.6	928.5	14.8
PBE0/6-311G(d,p)	3136.4	38.3	1327.1	14.7	932.5	10.7
PBE0/6-311+G(d,p)	3137.0	37.6	1326.1	15.7	932.1	11.2
PBE0/6-311++G(d,p)	3136.9	37.7	1326.1	15.7	932.1	11.2
PBE0/6-311G(2d,2p)	3148.7	26.0	1331.1	10.6	935.6	7.6
PBE0/6-311+G(2d,2p)	3149.9	24.7	1330.4	11.4	935.3	7.9
PBE0/6-311++G(2d,2p)	3149.9	24.8	1330.4	11.4	935.3	7.9
PBE0/6-311G(df,pd)	3145.6	29.1	1330.8	11.0	935.2	8.1
PBE0/6-311+G(df,pd)	3146.3	28.3	1329.9	11.9	934.8	8.5
PBE0/6-311G(2df,2pd)	3155.1	19.5	1334.0	7.8	937.6	5.7
PBE0/6-311+G(2df,2pd)	3156.1	18.5	1333.2	8.6	937.3	6.0
PBE0/6-311++G(2df,2pd)	3156.1	18.6	1333.2	8.6	937.3	6.0
PBE0/6-311G(3df,3pd)	3159.9	14.8	1335.2	6.6	938.6	4.6
PBE0/6-311+G(3df,3pd)	3161.0	13.7	1334.8	6.9	938.5	4.7
PBE0/6-311++G(3df,3pd)	3160.4	14.2	1334.8	6.9	938.5	4.8
PBE0/cc-pVDZ	3114.1	60.5	1320.0	21.8	927.1	16.2
PBE0/aug-cc-pVDZ	3116.2	58.4	1319.3	22.5	926.9	16.3
PBE0/cc-pVTZ	3152.9	21.7	1332.6	9.1	936.7	6.5
PBE0/aug-cc-pVTZ	3153.5	21.1	1332.5	9.3	936.7	6.6
PBE0-D3/6-31G(d,p)	3123.0	51.6	1322.9	18.9	929.3	13.9
PBE0-D3/6-31+G(d,p)	3122.7	51.9	1321.2	20.6	928.4	14.8
PBE0-D3/6-31++G(d,p)	3122.6	52.0	1321.2	20.6	928.4	14.8
PBE0-D3/6-311G(d,p)	3135.0	39.6	1327.1	14.7	932.4	10.8
PBE0-D3/6-311+G(d,p)	3135.8	38.8	1326.1	15.7	932.0	11.2
PBE0-D3/6-311++G(d,p)	3135.6	39.0	1326.1	15.7	932.0	11.2
PBE0-D3/6-311G(2d,2p)	3147.4	27.2	1331.2	10.6	935.5	7.7
PBE0-D3/6-311+G(2d,2p)	3148.6	26.0	1330.4	11.4	935.2	8.0
PBE0-D3/6-311++G(2d,2p)	3148.6	26.0	1330.4	11.4	935.2	8.0
PBE0-D3/6-311G(df,pd)	3145.6	29.0	1330.8	11.0	935.2	8.0
PBE0-D3/6-311+G(df,pd)	3146.3	28.3	1329.8	12.0	934.8	8.4
PBE0-D3/6-311G(2df,2pd)	3155.1	19.5	1334.0	7.8	937.6	5.6

PBE0-D3/6-311+G(2df,2pd)	3156.1	18.5	1333.2	8.6	937.3	5.9
PBE0-D3/6-311++G(2df,2pd)	3156.1	18.5	1333.2	8.6	937.3	5.9
PBE0-D3/6-311G(3df,3pd)	3159.9	14.7	1335.2	6.6	938.6	4.6
PBE0-D3/6-311+G(3df,3pd)	3161.0	13.6	1334.8	7.0	938.5	4.7
PBE0-D3/6-311++G(3df,3pd)	3160.4	14.2	1334.8	7.0	938.5	4.7
PBE0-D3/cc-pVDZ	3114.1	60.5	1320.0	21.8	927.1	16.1
PBE0-D3/aug-cc-pVDZ	3116.2	58.4	1319.3	22.5	926.9	16.3
PBE0-D3/cc-pVTZ	3152.9	21.7	1332.6	9.2	936.7	6.5
PBE0-D3/aug-cc-pVTZ	3153.5	21.1	1332.4	9.4	936.7	6.5
MN15/6-31G(d,p)	3184.8	–10.1	1339.9	1.9	943.1	0.1
MN15/6-31+G(d,p)	3183.8	–9.2	1338.3	3.5	942.2	1.0
MN15/6-31++G(d,p)	3183.8	–9.1	1338.3	3.5	942.2	1.0
MN15/6-311G(d,p)	3198.2	–23.6	1344.5	–2.8	946.6	–3.3
MN15/6-311+G(d,p)	3198.4	–23.7	1343.7	–2.0	946.2	–3.0
MN15/6-311++G(d,p)	3198.3	–23.7	1343.7	–1.9	946.2	–3.0
MN15/6-311G(2d,2p)	3210.7	–36.1	1349.0	–7.3	949.9	–6.7
MN15/6-311+G(2d,2p)	3211.2	–36.6	1348.3	–6.5	949.6	–6.4
MN15/6-311++G(2d,2p)	3211.2	–36.6	1348.3	–6.5	949.6	–6.4
MN15/6-311G(df,pd)	3210.9	–36.3	1349.1	–7.3	949.9	–6.7
MN15/6-311+G(df,pd)	3211.1	–36.5	1348.1	–6.4	949.5	–6.3
MN15/6-311G(2df,2pd)	3218.5	–43.9	1351.8	–10.0	952.0	–8.7
MN15/6-311+G(2df,2pd)	3219.6	–45.0	1351.2	–9.5	951.8	–8.6
MN15/6-311++G(2df,2pd)	3219.6	–45.0	1351.2	–9.5	951.8	–8.6
MN15/6-311G(3df,3pd)	3224.4	–49.8	1353.5	–11.7	953.3	–10.1
MN15/6-311+G(3df,3pd)	3224.4	–49.7	1353.0	–11.2	953.1	–9.8
MN15/6-311++G(3df,3pd)	3224.4	–49.8	1353.0	–11.2	953.1	–9.8
MN15/cc-pVDZ	3176.4	–1.7	1336.9	4.9	940.9	2.3
MN15/aug-cc-pVDZ	3177.4	–2.8	1337.2	4.6	941.1	2.1
MN15/cc-pVTZ	3219.2	–44.6	1351.5	–9.7	951.9	–8.7
MN15/aug-cc-pVTZ	3220.8	–46.2	1352.5	–10.8	952.5	–9.3
CAM-B3LYP-D3BJ/6-311G(d,p)	3202.4	–27.8	1345.5	–3.7	947.4	–4.2
CAM-B3LYP-D3BJ/6-311+G(d,p)	3202.8	–28.2	1344.6	–2.8	947.0	–3.8
CAM-B3LYP-D3BJ/6-311++G(d,p)	3202.7	–28.1	1344.6	–2.8	947.0	–3.8
CAM-B3LYP-D3BJ/cc-pVDZ	3179.0	–4.4	1337.5	4.2	941.4	1.8
CAM-B3LYP-D3BJ/aug-cc-pVDZ	3181.4	–6.8	1337.2	4.6	941.5	1.8
CAM-B3LYP-D3BJ/cc-pVTZ	3221.3	–46.6	1351.5	–9.7	952.0	–8.8
CAM-B3LYP-D3BJ/aug-cc-pVTZ	3221.9	–47.3	1351.3	–9.5	952.0	–8.8
CCSD/cc-pVDZ	3124.9	49.7	1318.5	23.2	927.3	16.0
Experimental (X_0)	3174.6		1341.8		943.2	

Table S2. Geometry parameters in the principal axes of inertia of benzothiazole calculated at the MP2/6-31G(d,p), MP2/6-311++G(d,p), and B3LYP-D3BJ/6-311++G(d,p) levels of theory. The atoms are numbered according to Figure 1.

	MP2/6-31G(d,p)			MP2/6-311++G(d,p)			B3LYP-D3BJ/6-311++G(d,p)		
	a /Å	*b* /Å	*c* /Å	*a* /Å	*b* /Å	*c* /Å	*a* /Å	*b* /Å	*c* /Å
S1	1.75670	0.90336	0.00006	1.79115	0.94210	–0.00043	–1.78438	0.93513	–0.00004
C2	2.05502	–0.81055	–0.00009	2.10968	–0.79468	0.00034	–2.11102	–0.77361	0.00011
N3	1.01250	–1.59911	0.00001	1.08179	–1.57085	–0.00002	–1.08169	–1.57932	0.00000
C4	–0.14355	–0.83554	0.00001	–0.09354	–0.83246	0.00001	0.08684	–0.83498	–0.00001
C5	–1.45184	–1.35574	0.00003	–1.38598	–1.36934	0.00006	1.38634	–1.37675	–0.00004
C6	–2.52439	–0.47098	0.00004	–2.47086	–0.50613	0.00019	2.47338	–0.50986	–0.00006
C7	–2.31345	0.92663	–0.00003	–2.28560	0.88608	0.00023	2.28559	0.89105	0.00001
C8	–1.02856	1.46307	–0.00002	–1.01194	1.44000	0.00015	1.00976	1.44868	0.00001
C9	0.05722	0.56993	–0.00009	0.07963	0.57115	0.00004	–0.09065	0.57362	0.00009
H10	3.07678	–1.17559	–0.00013	3.13339	–1.14614	0.00029	–3.13868	–1.12169	0.00016
H11	–1.59591	–2.43233	0.00007	–1.51298	–2.44467	0.00004	1.51258	–2.45558	–0.00008
H12	–3.54025	–0.85605	0.00006	–3.47666	–0.90870	0.00023	3.48273	–0.91168	–0.00009
H13	–3.16880	1.59683	–0.00001	–3.14929	1.54035	0.00027	3.15192	1.54701	–0.00002
H14	–0.87295	2.53812	–0.00005	–0.87377	2.51393	0.00008	0.87196	2.52616	0.00004

Table S3a. Calculated (ν_{calc}) and observed (ν_{obs}) frequencies of the main species of benzothiazole. $\nu_{obs} - \nu_{calc}$ values are obtained after a fit with the program *XIAM*. J, K_a, and K_c are the symmetric top rotational quantum numbers. F is the total angular momentum in the coupled basis with $F = J + I$.

N°	J	K_a	K_c	F	J	K_a	K_c	F	ν_{obs} / MHz	$\nu_{obs} - \nu_{calc}$ / kHz
1	1	1	1	2	0	0	0	1	4118.0679	1.5
2	1	1	1	1	0	0	0	1	4116.8446	–0.2
3	1	1	1	0	0	0	0	1	4119.8990	0.3
4	2	0	2	3	1	0	1	2	4511.8047	–0.2
5	2	0	2	2	1	0	1	1	4511.6282	–0.4
6	2	0	2	1	1	0	1	0	4512.3594	2.8
7	2	2	1	3	1	1	0	2	10467.3588	1.7
8	2	2	1	2	1	1	0	2	10466.8420	–0.1
9	2	2	1	2	1	1	0	1	10466.1020	0.8
10	2	2	1	1	1	1	0	1	10466.9017	–0.5
11	2	2	1	1	1	1	0	0	10468.7544	–0.2
12	2	2	0	3	1	1	1	2	10923.7182	1.5
13	2	2	0	2	1	1	1	2	10923.4116	–0.8
14	2	2	0	2	1	1	1	1	10924.6342	0.2
15	2	2	0	1	1	1	1	1	10925.1068	–0.7
16	2	2	0	1	1	1	1	0	10922.0533	–0.3
17	2	2	1	3	2	1	2	3	6694.1269	0.4
18	2	2	1	3	2	1	2	2	6694.9220	1.7
19	2	2	1	2	2	1	2	3	6693.6114	–0.1
20	2	2	1	2	2	1	2	2	6694.4049	–0.5
21	2	2	1	2	2	1	2	1	6693.1715	1.0
22	2	2	1	1	2	1	2	2	6695.2059	–0.5
23	2	2	1	1	2	1	2	1	6693.9715	0.0
24	3	1	3	4	2	1	2	3	6223.1441	0.1
25	3	1	3	3	2	1	2	2	6223.1862	0.1
26	3	1	3	2	2	1	2	1	6222.9633	–2.7
27	3	1	3	4	2	0	2	3	7715.9238	0.4
28	3	1	3	3	2	0	2	3	7715.1718	0.2
29	3	1	3	3	2	0	2	2	7714.8676	0.3
30	3	1	3	2	2	0	2	1	7716.3562	0.5
31	3	2	2	4	2	2	1	3	6854.8730	–1.2
32	3	2	2	3	2	2	1	2	6855.3861	–3.0
33	3	2	2	2	2	2	1	1	6854.5878	–0.3
34	3	2	2	4	2	1	1	3	12353.8733	0.3
35	3	2	2	3	2	1	1	2	12352.5617	–2.5
36	3	2	2	2	2	1	1	1	12354.6008	0.7
37	3	2	1	4	2	1	2	3	13832.0963	1.1

38	3	2	1	3	2	1	2	3	13832.6108	–1.6
39	3	2	1	3	2	1	2	2	13833.4059	–0.4
40	3	2	1	2	2	1	2	2	13832.7074	–0.7
41	3	2	1	2	2	1	2	1	13831.4725	–0.7
42	3	3	0	4	2	2	1	3	17055.1071	0.8
43	3	3	0	3	2	2	1	3	17054.5143	–1.9
44	3	3	0	3	2	2	1	2	17055.0305	–0.6
45	3	3	0	2	2	2	1	2	17055.8256	–2.2
46	3	1	2	4	3	0	3	4	3471.1594	1.0
47	3	1	2	4	3	0	3	3	3471.1370	–4.6
48	3	1	2	3	3	0	3	3	3472.5544	–0.6
49	3	1	2	2	3	0	3	2	3470.6697	0.0
50	3	2	2	4	3	1	3	4	7325.8561	–0.6
51	3	2	2	3	3	1	3	3	7326.6055	–2.9
52	3	2	2	2	3	1	3	2	7325.5930	–0.6
53	3	3	0	4	3	2	1	4	9917.1386	1.0
54	3	3	0	4	3	2	1	3	9916.6213	1.0
55	3	3	0	3	3	2	1	4	9916.5462	–1.2
56	3	3	0	3	3	2	1	3	9916.0298	–0.4
57	3	3	0	3	3	2	1	2	9916.7313	2.9
58	3	3	0	2	3	2	1	3	9916.8240	–2.9
59	3	3	0	2	3	2	1	2	9917.5238	–1.3
60	3	3	1	4	3	2	2	4	10194.8566	1.8
61	3	3	1	3	3	2	2	3	10194.2510	1.8
62	3	3	1	2	3	2	2	2	10195.0654	–1.3
63	4	0	4	5	3	1	3	4	7534.1955	–0.2
64	4	0	4	4	3	1	3	3	7534.6401	1.3
65	4	0	4	3	3	1	3	2	7534.0146	2.7
66	4	1	3	5	3	1	2	4	9803.2122	0.7
67	4	1	3	4	3	1	2	3	9803.1649	0.2
68	4	1	3	3	3	1	2	2	9803.3542	–0.6
69	4	1	4	5	3	1	3	4	8240.9492	–0.1
70	4	1	4	4	3	1	3	3	8240.9039	1.5
71	4	1	4	3	3	1	3	2	8240.8899	–1.7
72	4	2	2	5	3	2	1	4	9613.9025	–0.1
73	4	2	2	4	3	2	1	3	9614.4051	0.1
74	4	2	2	3	3	2	1	2	9613.8223	0.9
75	4	2	3	5	3	2	2	4	9094.4321	1.2
76	4	2	3	4	3	2	2	3	9094.5991	2.4
77	4	2	3	3	3	2	2	2	9094.3887	0.4
78	4	3	1	5	3	3	0	4	9273.6481	–0.5
79	4	3	1	4	3	3	0	3	9274.2603	0.0
80	4	3	1	3	3	3	0	2	9273.4363	–0.3
81	4	3	1	5	3	2	2	4	19473.8834	2.6
82	4	3	1	4	3	2	2	3	19473.9081	5.9

83	4	3	1	3	3	2	2	2	19473.8727	–2.6
84	4	2	2	5	4	1	3	5	5034.3772	1.1
85	4	2	2	4	4	1	3	4	5034.0294	0.2
86	4	2	2	3	4	1	3	3	5034.4654	0.0
87	5	0	5	6	4	0	4	5	10512.3878	0.1
88	5	0	5	5	4	0	4	4	10512.0977	–1.0
89	5	0	5	4	4	0	4	3	10512.4276	–2.6
90	5	0	5	6	4	1	4	5	9805.6339	–0.3
91	5	0	5	5	4	1	4	4	9805.8352	0.2
92	5	0	5	4	4	1	4	3	9805.5512	0.7
93	5	1	4	6	4	1	3	5	12108.2372	0.6
94	5	1	4	5	4	1	3	4	12108.0896	–0.8
95	5	1	4	5	4	1	3	5	12109.4566	–0.4
96	5	1	4	4	4	1	3	3	12108.3399	0.5
97	5	1	5	6	4	1	4	5	10223.5744	0.2
98	5	1	5	5	4	1	4	4	10223.4977	0.7
99	5	1	5	4	4	1	4	3	10223.5461	–1.1
100	5	1	5	6	4	0	4	5	10930.3283	0.5
101	5	1	5	5	4	0	4	4	10929.7595	–1.2
102	5	1	5	4	4	0	4	3	10930.4237	–3.1
103	5	2	3	6	4	2	2	5	12205.1819	1.0
104	5	2	3	5	4	2	2	4	12205.5047	0.3
105	5	2	3	4	4	2	2	3	12205.1684	–1.1
106	5	2	4	6	4	2	3	5	11295.8664	0.7
107	5	2	4	5	4	2	3	4	11295.8981	1.5
108	5	3	3	6	4	3	2	5	11574.9128	–0.9
109	5	3	3	5	4	3	2	4	11575.2111	–0.8
110	5	3	3	4	4	3	2	3	11574.8500	–0.6
111	5	3	3	6	4	2	2	5	21114.6092	0.7
112	5	3	3	5	4	2	2	4	21113.8427	–1.0
113	5	3	3	4	4	2	2	3	21114.8178	–1.0
114	6	0	6	7	5	0	5	6	12363.0622	–0.5
115	6	0	6	6	5	0	5	5	12362.8427	–0.4
116	6	0	6	5	5	0	5	4	12363.0808	1.8
117	6	0	6	7	5	1	5	6	11945.1229	0.2
118	6	0	6	6	5	1	5	5	11945.1810	–0.2
119	6	0	6	5	5	1	5	4	11945.0827	0.4
120	6	1	6	7	5	1	5	6	12174.8894	0.9
121	6	1	6	6	5	1	5	5	12174.8036	0.4
122	6	1	6	5	5	1	5	4	12174.8716	–0.8
123	6	1	6	7	5	0	5	6	12592.8290	0.5
124	6	1	6	6	5	0	5	5	12592.4651	–0.1
125	6	1	6	5	5	0	5	4	12592.8681	–1.0
126	6	2	4	7	5	2	3	6	14784.3563	–0.6
127	6	2	4	6	5	2	3	5	14784.5307	0.4

128	6	2	4	5	5	2	3	4	14784.3769	2.5
129	6	2	4	7	6	1	5	7	5621.0560	0.5
130	6	2	4	6	6	1	5	6	5621.5801	–0.5
131	6	2	4	5	6	1	5	5	5620.9651	–1.7
132	6	3	3	7	6	2	4	7	8443.9111	0.6
133	6	3	3	6	6	2	4	6	8443.1536	0.5
134	6	3	3	5	6	2	4	5	8444.0383	0.0
135	7	0	7	8	6	1	6	7	13981.8762	2.2
136	7	0	7	7	6	1	6	6	13981.8613	0.2
137	7	0	7	6	6	1	6	5	13981.8498	–2.4
138	7	1	7	8	6	0	6	7	14331.5410	–1.2
139	7	1	7	7	6	0	6	6	14331.3157	–0.1
140	7	1	7	6	6	0	6	5	14331.5564	1.7
141	7	3	4	8	6	3	3	7	16761.4171	1.2
142	7	3	4	7	6	3	3	6	16761.7264	1.2
143	7	3	4	6	6	3	3	5	16761.3874	–2.5
144	7	3	5	8	6	3	4	7	16210.8442	–1.1
145	7	3	5	7	6	3	4	6	16210.9314	–0.8
146	7	2	5	8	7	1	6	8	6576.2589	0.4
147	7	2	5	7	7	1	6	7	6577.1209	0.1
148	7	2	5	6	7	1	6	6	6576.1335	–0.6
149	7	2	6	8	7	1	7	8	11984.9376	0.9
150	7	2	6	7	7	1	7	7	11986.0628	0.1
151	7	2	6	6	7	1	7	6	11984.7745	0.2
152	7	3	5	8	7	2	6	8	11724.7717	0.3
153	7	3	5	7	7	2	6	7	11725.1989	0.3
154	7	3	5	6	7	2	6	6	11724.7101	0.3
155	7	4	3	8	7	3	4	8	13268.6735	0.1
156	7	4	3	7	7	3	4	7	13268.0038	–0.5
157	7	4	3	6	7	3	4	6	13268.7690	–0.8
158	8	0	8	9	7	1	7	8	15951.3896	2.4
159	8	0	8	8	7	1	7	7	15951.3462	1.5
160	8	0	8	7	7	1	7	6	15951.3695	–4.0
161	8	1	8	9	7	0	7	8	16131.5730	0.1
162	8	1	8	8	7	0	7	7	16131.4287	0.7
163	8	2	6	9	7	3	5	8	12755.3359	–0.8
164	8	2	6	8	7	3	5	7	12756.3421	–0.4
165	8	2	6	7	7	3	5	6	12755.2192	–0.2
166	8	1	7	9	8	0	8	9	12491.0778	0.7
167	8	1	7	8	8	0	8	8	12492.5642	–0.5
168	8	1	7	7	8	0	8	7	12490.8898	0.0
169	8	3	5	9	8	2	6	9	7637.9407	–0.6
170	8	3	5	8	8	2	6	8	7637.7623	0.4
171	8	3	5	7	8	2	6	7	7637.9653	1.4
172	8	3	6	9	8	2	7	9	12596.6194	0.8

173	8	3	6	8	8	2	7	8	12597.1745	–0.1
174	8	3	6	7	8	2	7	7	12596.5481	–0.5
175	8	4	4	9	8	3	5	9	12605.5233	0.1
176	8	4	4	8	8	3	5	8	12604.8111	–0.3
177	8	4	4	7	8	3	5	7	12605.6122	–0.7
178	9	0	9	10	8	1	8	9	17881.3569	1.5
179	9	0	9	9	8	1	8	8	17881.3056	1.4
180	9	0	9	8	8	1	8	7	17881.3439	–1.4
181	9	1	9	10	8	0	8	9	17971.1238	–1.6
182	9	1	9	9	8	0	8	8	17971.0265	–0.4
183	9	4	5	10	9	3	6	10	11784.6518	0.8
184	9	4	5	9	9	3	6	9	11783.9679	–0.5
185	9	4	5	8	9	3	6	8	11784.7271	–0.2
186	10	0	10	11	9	1	9	10	19789.6560	1.2
187	10	0	10	10	9	1	9	9	19789.6061	1.7
188	10	0	10	9	9	1	9	8	19789.6456	–0.9
189	10	1	10	11	9	0	9	10	19833.2605	–3.1
190	10	1	10	10	9	0	9	9	19833.1957	3.8
191	11	1	11	12	10	0	10	11	21707.4399	–3.0
192	11	1	11	11	10	0	10	10	21707.3905	3.1
193	12	1	12	13	11	0	11	12	23587.7688	–6.5
194	12	1	12	12	11	0	11	11	23587.7357	5.4

Table S3b. Calculated (ν_{calc}) and observed (ν_{obs}) frequencies of the ^{34}S isotopologue of benzothiazole. $\nu_{obs} - \nu_{calc}$ values are obtained after a fit with the program *XIAM*. J, K_a, and K_c are the symmetric top rotational quantum numbers. F is the total angular momentum in the coupled basis with $F = J + I$.

N°	J	K_a	K_c	F	J	K_a	K_c	F	ν_{obs} / MHz	$\nu_{obs} - \nu_{calc}$ / kHz
1	1	1	1	2	0	0	0	1	4073.8683	0.6
2	2	2	1	3	1	1	0	2	10361.2277	1.3
3	2	2	1	2	1	1	0	2	10360.7126	–0.3
4	2	2	1	2	1	1	0	1	10359.9724	0.8
5	2	2	1	1	1	1	0	1	10360.7682	–2.1
6	2	2	1	1	1	1	0	0	10362.6227	–0.9
7	2	2	0	3	1	1	1	2	10807.6730	2.2
8	2	2	0	2	1	1	1	2	10807.3659	0.8
9	2	2	0	2	1	1	1	1	10808.5854	–0.2
10	2	2	0	1	1	1	1	1	10809.0620	0.6
11	2	2	0	1	1	1	1	0	10806.0101	0.1
12	2	2	1	3	2	1	2	3	6640.9282	2.2
13	2	2	1	3	2	1	2	2	6641.7232	3.0
14	3	2	2	4	2	1	1	3	12221.2770	–0.2
15	3	2	2	3	2	1	1	2	12219.9660	–3.4
16	3	2	2	2	2	1	1	1	12222.0028	–0.9
17	5	0	5	6	4	1	4	5	9653.5289	–0.3
18	5	0	5	5	4	1	4	4	9653.7366	–0.1
19	5	0	5	4	4	1	4	3	9653.4461	1.8
20	5	1	5	6	4	0	4	5	10789.7675	–0.5
21	5	1	5	5	4	0	4	4	10789.1935	–1.6
22	5	1	5	4	4	0	4	3	10789.8652	–3.4
23	6	0	6	7	5	1	5	6	11767.3898	–0.1
24	6	0	6	6	5	1	5	5	11767.4526	–0.5
25	6	0	6	5	5	1	5	4	11767.3502	1.4
26	6	1	6	7	5	0	5	6	12425.9663	0.6
27	6	1	6	6	5	0	5	5	12425.5961	–0.9
28	6	1	6	5	5	0	5	4	12426.0040	–3.4
29	6	1	6	7	5	1	5	6	12002.0181	3.5
30	6	1	6	6	5	1	5	5	12001.9273	–2.6
31	6	1	6	5	5	1	5	4	12001.9937	–4.7
32	6	4	2	7	6	3	3	7	13638.8364	1.0
33	6	4	2	6	6	3	3	6	13638.2268	–1.2
34	6	4	2	5	6	3	3	5	13638.9368	–1.1

35	7	0	7	8	6	1	6	7	13779.2490	4.0
36	7	0	7	7	6	1	6	6	13779.2357	0.8
37	7	0	7	6	6	1	6	5	13779.2181	–4.8
38	7	1	7	8	6	0	6	7	14137.1148	–2.7
39	7	1	7	7	6	0	6	6	14136.8890	1.8
40	7	1	7	6	6	0	6	5	14137.1326	1.8
41	7	1	6	8	6	2	5	7	13511.6022	–0.3
42	7	1	6	7	6	2	5	6	13512.1577	–0.3
43	7	1	6	6	6	2	5	5	13511.5269	0.6
44	7	2	6	8	7	1	7	8	11825.6479	0.8
45	7	2	6	7	7	1	7	7	11826.7744	0.2
46	7	3	5	8	7	2	6	8	11609.5692	–0.1
47	7	3	5	7	7	2	6	7	11609.9898	–2.1
48	7	3	5	6	7	2	6	6	11609.5099	1.5
49	7	4	3	8	7	3	4	8	13207.0602	0.2
50	7	4	3	7	7	3	4	7	13206.3977	–0.2
51	7	4	3	6	7	3	4	6	13207.1559	0.4
52	8	0	8	9	7	1	7	8	15723.7496	2.3
53	8	0	8	8	7	1	7	7	15723.7112	5.0
54	8	0	8	7	7	1	7	6	15723.7278	–5.6
55	8	1	8	9	7	0	7	8	15909.3661	1.0
56	8	1	8	8	7	0	7	7	15909.2152	–2.5
57	8	2	6	9	7	3	5	8	12445.8887	3.1
58	8	2	6	8	7	3	5	7	12446.8950	–1.4
59	8	2	6	7	7	3	5	6	12445.7646	–3.0
60	8	1	7	9	8	0	8	9	12281.0967	1.6
61	8	1	7	8	8	0	8	8	12282.5947	1.2
62	8	1	7	7	8	0	8	7	12280.9045	–1.9
63	8	2	7	9	8	1	8	9	13398.5487	2.7
64	8	2	7	8	8	1	8	8	13399.6477	–2.1
65	8	2	7	7	8	1	8	7	13398.4039	–3.1
66	8	3	6	9	8	2	7	9	12457.8089	1.5
67	8	3	6	8	8	2	7	8	12458.3582	–0.7
68	8	3	6	7	8	2	7	7	12457.7377	–0.3
69	8	4	4	9	8	3	5	9	12563.4442	0.4
70	8	4	4	8	8	3	5	8	12562.7348	–1.4
71	8	4	4	7	8	3	5	7	12563.5325	–0.4
72	9	0	9	10	8	1	8	9	17628.3241	2.2
73	9	0	9	9	8	1	8	8	17628.2758	4.4
74	9	0	9	8	8	1	8	7	17628.3103	–1.4
75	9	1	9	10	8	0	8	9	17721.3966	0.2
76	9	1	9	9	8	0	8	8	17721.2966	0.2
77	9	3	7	10	9	2	8	10	13522.3451	1.8

78	9	3	7	9	9	2	8	9	13522.9894	–1.6
79	9	3	7	8	9	2	8	8	13522.2701	–0.8
80	9	4	5	10	9	3	6	10	11759.5990	–1.3
81	9	4	5	9	9	3	6	9	11758.9182	2.4
82	9	4	5	8	9	3	6	8	11759.6788	2.0
83	10	0	10	11	9	1	9	10	19510.8552	1.0
84	10	0	10	10	9	1	9	9	19510.8108	6.8
85	10	0	10	9	9	1	9	8	19510.8457	–0.2
86	10	1	10	11	9	0	9	10	19556.3608	2.2
87	10	1	10	10	9	0	9	9	19556.2871	1.2
88	10	3	7	11	9	4	6	10	13022.9281	0.8
89	10	3	7	10	9	4	6	9	13023.9482	0.6
90	10	3	7	9	9	4	6	8	13022.8314	–0.2
91	11	1	11	12	10	0	10	11	21403.6364	–1.8
92	12	1	12	13	11	0	11	12	23257.2487	–6.7
